# Revision of the genus *Hoplodrina* Boursin, 1937 (Lepidoptera, Noctuidae, Xyleninae). I. *Hoplodrina octogenaria* (Goeze, 1781) and its sister species *H. alsinides* (Costantini, 1922) sp. rev. in Europe

**DOI:** 10.3897/zookeys.927.51142

**Published:** 2020-04-16

**Authors:** Peter Huemer, Jean Haxaire, Kyung Min Lee, Marko Mutanen, Oleg Pekarsky, Stefano Scalercio, László Ronkay

**Affiliations:** 1 Tiroler Landesmuseen Betriebsges.m.b.H., Naturwissenschaftliche Sammlungen, Krajnc-Str. 1, A-6060 Hall, Austria Tiroler Landesmuseen Betriebsges.m.b.H. Innsbruck Austria; 2 Le Roc, 47310 LaPlume, France Unaffiliated LaPlume France; 3 Department of Ecology and Genetics, University of Oulu, PO Box 3000, FI-90014, Oulu, Finland University of Oulu Oulu Finland; 4 Felsőerdősor u. 16-18, H-1068, Budapest, Hungary Unaffiliated Budapest Hungary; 5 Council for Agriculture Research and Economics, Research Centre for Forestry and Wood, Rende, Italy Council for Agriculture Research and Economics Rende Italy; 6 Hungarian Natural History Museum, Budapest, Hungary Hungarian Natural History Museum Budapest Hungary

**Keywords:** cryptic species, ddRAD sequencing, DNA barcoding, morphology, owlet moths

## Abstract

The taxonomic status of the European *Hoplodrina octogenaria* (Goeze, 1781) is discussed and its partly sympatric sister species, *Hoplodrina alsinides* (Costantini, 1922) **sp. rev.**, is separated and re-described based on morphological and molecular taxonomic evidence. The adults and their genitalia are illustrated and DNA barcodes, as well as genome-wide single nucleotide polymorphism data collected by fractional genome sequencing (ddRAD), of the two species are provided.

## Introduction

The genus *Hoplodrina* was separated from the giant clade of ‘caradrines’ (actually the tribe Caradrinini) by Boursin (1937) who first recognised that this group is rather remote taxonomically from the other large groups of the tribe (*Caradrina* Ochsenheimer, 1816; *Athetis* Hübner, [1821]; *Spodoptera* Guenée, 1852; *Stenodrina* Boursin, 1937; and *Stygiodrina* Boursin, 1937). Boursin stated that *Hoplodrina* possesses a number of shared features (“synapomorphies”) such as the similar habitus with less striking brown or grey coloration and uniform forewing pattern with well-developed and clearly outlined orbicular and reniform stigmata, and very characteristic male clasping apparatus. The similarly distinctive features of the everted vesica and the female antrum and appendix bursae were at that time unknown to him.

The genus *Caradrina* (s.l.) has been revised by Hacker (2004); the other large clades are still unrevised though a number of new *Athetis* species were described in the last two decades. For *Hoplodrina*, the last and only comprehensive checklist was published by Fibiger and Hacker (2007); it comprised 17 species and three subspecies. Subsequently, two new eastern Asiatic species of *Hoplodrina* were described from the *H. conspicua* (Leech, 1900) species-group by Ronkay et al. (2013; *H. persequora* Ronkay, Ronkay, Fu & Wu, 2013, and *H. vestigiosa* Ronkay, Ronkay, Fu & Wu, 2013) and four new species from the *H. implacata* (Wileman & West, 1929) species-group from Taiwan by Wu and Owada (2018; *H. cienensis* Wu & Owada, 2018, *H. obscura* Wu & Owada, 2018, *H. bunun* Wu & Owada, 2018, and *H. kononenkoi* Wu & Owada, 2018). The genus *Hoplodrina* has, therefore, the largest diversity in the western Palaearctic (as stated by Fibiger and Hacker, op. cit.) but there has also been a remarkable increase in the eastern Asiatic fauna.

The European *Hoplodrina* fauna is generally considered to be well-known as all but one species was described before the description of the genus. The only exception is *H. hesperica* Dufay & Boursin, 1960, which was recognised and described only following more intensive studies of the genitalia of the Noctuidae (s.l.). The six European species are characterised and illustrated in detail by Fibiger and Hacker in the *Noctuidae Europaeae* series (2007).

This view, however, seems to be erroneous. The molecular taxonomic (barcoding) studies of the Alpine Noctuidae fauna showed, rather surprisingly, a clear splitting of “*octogenaria*” (Huemer 2013, Huemer and Hebert 2016, Huemer et al. 2019). These investigations were made independently and almost at the same time by the research groups of Peter Huemer and Jean Haxaire, providing the same results. The subsequent morphological survey was continued by Oleg Pekarsky and László Ronkay which proved the existence of clearly recognisable differences in the genitalia of both sexes and also in the forewing shape and pattern, although the external morphological features show a certain overlap. The validity of the two distinct species was finally proven true by fractional genome sequencing (ddRAD) data.

The results inspired further studies of the entire genus and the morphological results were both surprising and convincing at the same time. The study of the *octogenaria*-like populations from the entire known range demonstrates that not only the south-western Mediterranean (Spanish, Portuguese and north-west African) “*octogenaria*” taxa are partly misinterpreted but that there is an undescribed *octogenaria*-like species in the Canary Islands (Tenerife) and that the Alpine twin species of *octogenaria* occurs not only in the Alps (France, Italy, Austria and Slovenia) but also in the eastern Carpathians, the Balkans, the southern Apennines, Crete and Cyprus. Moreover, there are unsolved taxonomic problems with practically all western Palaearctic species, therefore, a full, integrative revision of the genus is required.

The present paper is the first part of this revisional work which contains the re-description of the sister species of *H. octogenaria*, including the detailed comparison of *H. alsinides* (Costantini, 1922) sp. rev. with *H. octogenaria* and the historical information on the descriptions by Costantini in 1921 and 1922.

## Material and methods

### Morphology and material

Our study is based on more than 3000 specimens of the *Hoplodrina octogenaria* species-group. Most of the material was traditionally set and dried, although a few specimens are pinned but remain unset. Genitalia preparations followed standard techniques for Noctuoidea, including everting the vesica.

### Molecular analysis

#### DNA barcode sequencing and analysis

DNA barcode sequences of the mitochondrial COI gene – a 658 base-pair long segment of the 5’ terminus of the mitochondrial COI gene (*cytochrome c oxidase 1*) – were obtained from 269 specimens belonging to seven species of European *Hoplodrina*, including publicly available specimens from the Barcode of Life Data Systems (BOLD). DNA samples from dried legs were prepared according to prescribed standards using the high-throughput protocol of deWaard et al. (2008). Samples were processed at the Canadian Centre for DNA Barcoding (CCDB, Biodiversity Institute of Ontario, University of Guelph). Details of successfully sequenced voucher specimens, including complete geographic data and images, can be accessed in BOLD (Ratnasingham and Hebert 2007) in the public dataset “Lepidoptera of the Europe - *Hoplodrina*” dx.doi.org/10.5883/DS- DS-LEEUHOPL. GenBank accession numbers can be retrieved from the dataset.

Degrees of intra- and interspecific variation in the DNA barcode fragments were calculated under the Kimura 2 parameter (K2P) model of nucleotide substitution using analytical tools in BOLD systems v. 4.0 (http://www.boldsystems.org).

#### ddRAD library preparation and bioinformatics

We used genomic DNA (gDNA) aliquots that were extracted at the CCDB following laboratory protocols used routinely in CCDB as explained in deWaard et al. (2008). The quantity of gDNA extracts was checked using PicoGreen Kit (Molecular Probes). In order to reach sufficient gDNA quantity and quality, whole genome amplification was performed using REPLI-g Mini Kit (Qiagen) due to its low concentrations of gDNA in the original extracts. The ddRAD library was implemented following protocols described in Lee et al. (2018) with two exceptions: gDNA was digested with *Pst*I and *Msp*I and the size distribution was measured with Bioanalyzer (Agilen Technologies). The de-multiplexed fastq data are archived in the NCBI SRA: SRP155300.

Raw paired-end reads were de-multiplexed with no mismatches tolerated using their unique barcode and adapter sequences using *ipyrad* v.0.7.23 (Eaton and Overcast 2016). All *ipyrad* defaults were used, with the following exceptions: the minimum depth at which majority rule base calls are made was set to 3, the cluster threshold was set to 0.85, and the minimum number of samples with data for a given locus to be retained in the final data set was set to 2–4, and 13.

We then applied a number of filters to identify candidate diagnostic single nucleotid polymorphisms (SNPs) for detecting fixed allelic differences between two species. We focused on loci genotyped for all individuals assayed (0% missing data) and on ddRAD tags containing only one bi-allelic.

### Phylogenetic analysis

To study the phylogenetic relationships among taxa and to test the validity of prevailing species hypotheses, we conducted maximum likelihood (ML) trees. Phylogenetic trees were constructed for the concatenated ddRAD data. ML tree was inferred in RAxML v.8.2.0 (Stamatakis 2014) with bootstrap support estimated by a 1000 replicates rapid-bootstrap analysis from the un-partitioned GTR+CAT model. We visualized the resulting phylogeny and assessed bootstrap support using FigTree v.1.4.2 (Rambaut 2015).

### Examination of *Wolbachia* infection

In order to exclude the presence of the bacterial parasite *Wolbachia*, we sequenced two markers of *Wolbachia*, FstZ and Wsp using primers and laboratory procedures of Ivanov et al. (2018). None of the samples was *Wolbachia* infected.

### Abbreviations of private and institutional collections

**CJHL** Collection Jean Haxaire, Laplume, France

**CREA-FL** Centro di ricerca Foreste e Legno (Research Centre for Forestry and Wood), Rende, Italy

**HNHM** Hungarian Natural History Museum, Budapest, Hungary

**LMK** Landesmuseum Kärnten, Klagenfurt, Austria

**MCSN** Museo Civico di Storia Naturale, Milano, Italy

**MNHU** Museum für Naturkunde, Humboldt-Universität, Berlin, Germany

**NHM** The Natural History Museum (formerly British Museum, Natural History), London, United Kingdom

**NHMW** Naturhistorisches Museum Wien, Austria

**RNS** Royal Natural History Museum (Naturhistoriska Riksmuseet), Stockholm, Sweden

**TLMF** Tiroler Landesmuseum Ferdinandeum, Innsbruck, Austria

**ZMHU** Museum für Naturkunde – Leibniz-Institut für Evolutions- und Biodiversitätsforschung, Berlin, Germany

**ZSM** Zoologische Staatssammlung, Munich, Germany

## Historical interpretations of taxa in the *Hoplodrina octogenaria* (Goeze, 1781) species-group

According to the published checklist of Fibiger and Hacker (2007), the *octogenaria* species-group includes the following species:

– octogenaria (Goeze, 1781)
  = alsines (Brahm, 1791) (Phalaena Noctua)
  = sordida (Haworth, 1809) (praeocc.) (Noctua)
  = sericea (Speyer, 1867) (Caradrina)
  = alsinides (Costantini, 1922) (Caradrina)
  = melendezi Agenjo, 1941;
– octogenaria ssp. amurensis (Staudinger, 1892) (Caradrina)
– pfeifferi (Boursin, 1932) (Athetis)
– blanda ([Denis & Schiffermüller], 1775) (Noctua)
– blanda ssp. robusta Boursin, 1940 (Hoplodrina)
– hesperica Dufay & Boursin, 1960
– levis (Staudinger, 1888) (Caradrina)
– straminea (Zerny, 1934) (Athetis)

They noted that “The priority of *octogenaria* (Goeze, 1781) over *alsines* Brahm, 1791 was introduced by Koçak (1983). The taxon *amurensis* (Staudinger, 1892) was originally described as subspecies of “*Caradrina alsines*”, later on treated by Boursin as “ ‘bona species’ (cf. Boursin’s never published systematic lists of Palaearctic trifine Noctuidae). Today in both standard publications on the eastern Palaearctic fauna: Kononenko et al. (1998), and Kononenko (2003, 2005) *amurensis* is suggested to be the eastern subspecies of *octogenaria*”.

Most of the information presented in “Noctuidae Europaeae” (Volume 9) (Fibiger and Hacker 2007) seems to be correct, except the following statements:

– melendezi is not synonymous with octogenaria but a distinct taxon. Further investigations of the Iberian “octogenaria” populations are needed to clarify whether melendezi represents a distinct species comprising different subspecies or are there numerous subspecies of octogenaria that occur in Spain and Portugal;
– pfeifferi includes more than one closely related species;
– blanda, hesperica and levis represent a distinct species-group while straminea is a member of the octogenaria species-complex;
– the species occurring in Asia Minor and often called “levis” represents another, still undescribed, species; and
– amurensis is a species distinct from octogenaria.

### The *Caradrina alsinides* problem

After the determination of the sister-species relationship of the two ‘*octogenaria*’ species, the next major problem was to clarify the identity of the described taxa formerly considered as synonymous with *octogenaria*. The problem was rather difficult as 1) the type material of the historical taxa is generally inaccessible; 2) the type localities of *octogenaria* and *alsines* are not stated.

Our concept was that although the types of the taxa described by Goeze and Brahm are not available and their type localities are not stated, their descriptions by inference refer to taxa that occur in Germany. Checking a considerable amount of material of *octogenaria* from different parts of Europe resulted in no specimens of the second species being found from Germany and north or north-west of the Alps. Thus, we treat *octogenaria* as a widespread European species occurring also in northern and north-western Europe. The neotype of *H. octogenaria* is designated below and illustrated in Fig. 11.

As the type-locality of *sordida* (a preoccupied name) is England, and that of *sericea* is Amsterdam, only *alsinides* (Costantini, 1921/1922) remained as a possible candidate for the sister species of *octogenaria*. This species was described from northern Italy (Prov. Modena: Mutina, Sestola), the region where the two species may occur sympatrically.

The case of *alsinides* therefore seemed rather difficult, especially on the assumption that the types were missing. The species was described twice by Alessandro Costantini, first in 1921, providing only the name without any additional information; therefore, the name can be considered as nomen nudum. A year later, he published a Latin description of the species mentioned first in 1921; therefore, the date of the valid description is in reality 1922 (ICZN 1999). The description is brief, without illustrations but includes mention of external features which help towards determination: “*Caradrina alsinides* m., n. species: (*) *C. alsinii* affinis, sed alis vero amplioribus, alar. ant. mac. cellularib. majoribus, orbiculari perfecter rotunda, ambabus fusco-repletis et valde appariscentibus; caterum haec species tam similis est ad alsinem Brahm, quam sp. taraxaci Hb. (*blanda* Tr., nec Hb.) ad superstem Tr. similiat!” The translation of the Latin text is as follows: “*Caradrina alsinides* m[ihi]., n. species: (*) similar to *C* (*aradrina*) *alsines*, but with broader wings, larger cellular maculae, perfectly rounded orbicular stigma, conspicuous and uniformly dark filling of stigmata; this species is as similar to *alsines* Brahm as to *taraxaci* Hb. (*blanda* Tr., nec Hb.) and *superstes* Tr.!”

The description expressly states that *alsinides* differs from *alsines* by its broader wings, larger and darker filled stigmata and perfectly rounded orbicular stigma. These characters are typical of *octogenaria* while its sister species has somewhat narrower forewings, less conspicuous and usually smaller or significantly smaller stigmata and the orbicular stigma is often somewhat flattened. However, based solely on this description the identity of the species, remained doubtful. We therefore tried to obtain syntype material and finally succeeded with a request to the Museo Civico di Storia Naturale, Milano. The male syntype, illustrated in Fig. 16, is designated below as the lectotype. With support from Axel Hausmann (ZSM) this specimen was successfully sequenced with NGS protocols. The sequencing provided, however, a surprising result as the DNA barcode of the lectotype specimen is identical with that of the sister species of *H. octogenaria*, despite the different external characters of the lectotype specimen and the differently stated features provided by the original description. This fact emphasizes the need of the genitalia and molecular investigations during the identification of the southern European “*octogenaria*-like” specimens.

The consequence of our investigations is that, surprisingly, *Hoplodrina alsinides* is a cryptic species in central and southern Europe. The re-description, and the detailed comparison with its sister species, *H. octogenaria*, is provided below.

## Systematic results

### Hoplodrina octogenaria

Taxon classificationAnimaliaLepidopteraNoctuidae

(Goeze, 1781)

3540032F-079A-5F5F-A0F2-2997C2DC4AE5

Figs 11–20 , 23 , 24 , 27 , 28

Phalaena Noctua octogenaria Goeze, 1781, Entomologische Beyträge zu des Ritter Linné zwölften Ausgabe des Natursystems 3(3): 227. Type-locality: Germany, Bayern, Landshut. Neotype: male, in coll. TLMF, here designated.
Phalaena Noctua alsines Brahm, 1791, Handbuch der Ökonomischen Insektengeschichte in Form eines Kalenders bearbeitet 2: 114. Type-locality: no locality given [Germany];
Noctua sordida Haworth, 1809, Lepidoptera Britannica; sistens Digestionem novam Insectorum Lepidopterorum quae in Magna Britannia Reperiuntur, Larvarum Pabulo, Temporeque Pascendi; Expansione Alarum; Mensibusque Volandi; Synonymis atque Locis Observationibusque Variis 2: 207. Type-locality: Great Britain;
Caradrina sericea Speyer, 1867, Entomologische Zeitung herausgegeben von dem Entomologischen Vereine zu Stettin 28: 73. Type-locality: [Netherlands] Amsterdam;

#### Neotype designation.

Neotype of *Phalaena Noctuaalsines* Brahm, 1791 (Fig. 11): Male, “Landshut/Bay. | Roβberg | 1.7.1972 | coll.-Nr. 960C | Reiser”, “TLMF | Innsbruck | H.Kolbeck | 2014-032” (coll. TLMF).

### Hoplodrina alsinides

Taxon classificationAnimaliaLepidopteraNoctuidae

(Costantini, 1922) sp. rev.

367E04E1-9F65-5FE9-911E-62D87311ECCE

Figs 1–10 , 21 , 22 , 25 , 26

Caradrina alsinides Costantini, 1922, Neue Beiträge zur systematischen Insektenkunde 2: 98. Type-locality: Italy, Prov. Modena (Emilia Romagna), Sestola. Lectotype: male, here designated.

#### Lectotype designation.

Lectotype of *Caradrina alsinides* Costantini, 1922 (Fig. 16): Male, “Emilia | Sestola | 21.VII.905 | A. Fiori”, “♂” “BC ZSM Lep 106561” (coll. MCSN).

#### Additional material examined.

**Spain**. 1 female, Aragon, Canfranc-Estacion, 1320 m, 42°45.73'N, 0°30.48'W, 13.VII.2012, leg. P. Huemer, TLMF 2013-013 (TLMF).

**France**. 1 female, Dep. Alpes-Maritimes, St. Martin Vesubie, 22.VII.1925, leg. A. Schmidt (HNHM); 1 female, Dep. Alpes-Maritimes, Col de la Cayolle, 2000 m, 6–13.VIII.1972, leg. R. Schütz, TLMF Innsbruck H. Kolbeck 2014-032 (TLMF); 1 male, Alpes-Maritimes, Col de la Couillole, 1600 m, 13.VII.1972, leg. M. Tarrier (TLMF); 1 female, Pyrénées Orientales, Road from Py to Mantet, 1704 m, 10.VII.1999, 42°29'03.68"N, 2°18'55.73"E, leg. J. Haxaire & O. Paquit [CJHL]; 1 male, from the same site, 21.VII.2001, leg. J. Haxaire & O. Paquit, BC-HAXNoctu0522 (barcode) [CJHL]; 2 males, from the same site, 29.VIII.2011, leg. J. Haxaire & O. Paquit [CJHL]; 4 males, 1 female, from the same site, 10.VII.2018, leg. J. Haxaire & M. Colin [CJHL]; 2 females, Pyrénées Orientales, Road from Py to Mantet, col de Mantet, 1764 m, 13.VII.2018, 42°28'52.15"N, 2°18'47.61"E, leg. J. Haxaire & O. Paquit [CJHL]; 3 males, Pyrénées Orientales, « refuge de Mariailles », trail to the Pla Guillem, 1752 m, 9.VIII.1997, 49°29'52.63"N, 2°24'33.47"E, leg. J. Haxaire & P. Beguin [CJHL]; 1 female, from the same site, 12.IX.1999, 49°29'52.63"N, 2°24'33.47"E, leg. J. Haxaire & O. Paquit [CJHL]; 1 male, from the same site, 23.VIII.2000, 49°29'52.63"N, 2°24'33.47"E, leg. J. Haxaire & O. Paquit, BC-HAXNoctu0520 (barcode) [CJHL].

**Switzerland**. 1 male, 6 females, Wallis, Simplon, Gabi, 7–10.VII.1968, leg. J. Wettstein (HNHM); 1 male, Wallis, Zermatt, 13.VII.1968, leg. J. Wettstein (HNHM); 1 female, Ticino, Mergoscia, 10.VIII.1971, leg. R. Müller (TLMF).

**Italy**. 2 males, Prov. South Tyrol, 7–10.VII.2004, leg. L. Ronkay & A. Kun (coll. HNHM); 2 males, 3 females, Prov. South Tyrol, Sesvenna Mts, above Prämajur, Watles, 1850 m, 18.VII.2006, leg. L. Ronkay & M. Tóth-Ronkay (HNHM and coll. G. Ronkay); 3 females, Prov. South Tyrol, Sesvenna Mts., Prämajur, above Lutaschg, 1700 m, 17.VII.2006, leg. L. Ronkay & M. Tóth-Ronkay (HNHM and coll. G. Ronkay); 1 male, Prov. South Tyrol, Vinschgau, Schleis, Schleiser Leiten, 1350 m, 46°41'517"N, 10°30'59"E, 5.VII.2013, leg. P. Huemer, TLMF 2013-013 (TLMF); 1 female, Prov. South Tyrol, Schnals, Fuchsberg, 1080 m, 46°40'27"N, 10°56'42"E, 7.VII.2014, leg. P. Huemer (TLMF); 1 male, South Tyrol, Ritten, Obergrünwald, 1750 m, 19.VII.2010, leg. P. Huemer, slide No. RL10288m (TLMF); 1 male, South Tyrol, Ritten, Obergrünwald, 1750 m, 19.VII.2010, leg. Peter Huemer, slide No. RL10289m; DNA Barcode ID TLMF Lep 02472 (TLMF); 2 males, Prov. South Tyrol, St. Felix, Warmesbrunn, 1500 m, 46°29'20"N, 11°09'27"E, 27.VI.2014, leg. S. Erlebach, TLMF 2014-001 (TLMF); 3 males, 5 females, South Tyrol, Mendel, Umg. Penegal, 1690 m, 46°26'13"N, 11°12'58"E, 22.VII.2019, leg. Huemer, TLMF Lep 27814, 27815 (barcodes) (TLMF); 1 female, South Tyrol, St. Ulrich, Ende Juli 1911, No. 7804, coll. J. Sterneck, slide No. RL12123f (NHMW); 2 males, Prov. Trento, Travignolo valley, Paneveggio, 1500 m, 31.VII., leg. F. Daniel (HNHM); 1 female, Prov. Trento, Madonna di Campiglio, campo Colf, 1650 m, 19.VII.1939, leg. A. Schmidt (coll. HNHM); 1 male, 1 female, Prov. Cuneo, Entracque S, Vallone di Moncolomb, 1430 m, 44°09'30"N, 7°24'0"E, 23.VII.2008, leg. M. Kahlen, TLMF 2009-027, slide Nos OP1413m, OP1414f (TLMF); 1 male, Prov. Cuneo, Demonte NW, Colle Valcavera NE, 2420 m, 44°23'04"N, 7°06'23"E, 17.VIII.2012, leg. P. Huemer, TLMF 2013-013 (TLMF); 1 male, Prov. Cuneo, N Colle della Lombarda, 1750 m, 44°15'08"N, 7°06'32"E, 17.VII.2012, leg. P. Huemer, TLMF 2013-013 (TLMF); 2 males, 4 females, Prov. Torino, PN Orsiera – Rocciavrè, Fenestrelle, ca. 1 km WNW Pequerel, 1840 m, 45°2'59"N, 7°3'5"E, 28.VI.2019, leg. Huemer (TLMF); 2 males, 2 females, Prov. Torino, PN Orsiera – Rocciavrè, Fenestrelle, ca. 0.7 km NE Pequerel, 1820 m, 45°3'6"N, 7°4'16"E, 23.VII.2019, leg. Huemer TLMF, Lep 27809 (barcode) (TLMF); 3 males, 6 females, Prov. Torino, PN Orsiera – Rocciavrè, Usseaux, Colle delle Finestre N, 2180 m, 45°4'21"N, 7°3'11"E, 24.VII.2019, TLMF Lep 27808, 27810 (barcodes) leg. Huemer (TLMF); 1 male, Prov. Chieti, PN della Majella, vic. of Blockhaus, ca. 2100 m, 42°08'48"N, 14°16'43"E, 22.VII.2011, leg. P. Huemer, TLMF 2012-010, slide No. OP1417m, BC TLMF Lep 05904 (barcode) (TLMF); 1 female, Prov. Chieti, Taranta Peligna, Pian di Valle, 1400 m, 42°02'19"N, 14°09'11"E, 21.VII.2011, leg. P. Huemer, TLMF 2012-010, slide No. OP1418f, BC TLMF Lep 06042 (barcode) (TLMF); 1 male, Calabria, Sila, Vivaio Sbanditi (CS), 1355 m, 39°23'30"N, 16°36'08"E, 17.VII.2014, leg. S. Scalercio; 1 male, from the same site, 29.VII.2014, leg. Scalercio & Infusino; 1 female, Calabria, Sila, Vivaio Sbanditi (CS), 1350 m, 39.3889N, 16.6022E, 6.VII.2015, leg. Scalercio & Infusino; 2 males, Calabria, SL_C2, Colle Macchie, Pedace (CS), 1450 m, 39.2597N, 16.5308E, 17.VII.2015, leg. Scalercio & Infusino; 2 males, Calabria, SPSE, Loc. Spinarva-Taverna (CZ), 1570 m, 39.0900N, 16.6800E, 27.VII.2017, leg. Scalercio & Infusino; 1 male, Calabria, SL_B1, Sila, Torre Scarda (CS), 1340 m, 39.2384N, 16.5131E, 17.VII.2015, leg. Scalercio & Infusino, LEP-SS-00439 (barcode) (CREA-FL); 1 ex., Calabria, Sila grd., La Fossita, 1305 m, 13.VII.2013, leg. Hausmann, BC ZSM lep 92607 (barcode) (ZSM).

**Austria**. 1 male, Styria, Prebichl, Reichenstein, 20.VII.1938, leg. Dr Szabó (HNHM); 1 male, Kals, 26.VII.1937, leg. Dr Szabó (HNHM); 1 female, Styria, Dürriegel, 21.VII.1917, coll. Dr Galvagni, slide No. RL12127f (NHMW); 1 female, Styria, Sausalgebirge, Kitzeck, 300–500 m, 3–9.VIII.1954, leg. F. Daniel (HNHM); 1 female, Styria, NP Gesäuse, Wagriedschlag SW Hieflau, 1450 m, 18.VII.2015, leg. H. Habeler, TLMF Innsbruck Slg. H. Habeler 2017-010 (TLMF); 1 male, Carinthia, Naggl, 13.VII.1934, coll. Dr Galvagni, slide No. RL12122m (NHMW); 1 male, Styria, Dfstr., Aflenz, 1882, slide No. RL12126m (NHMW); 1 male, Carinthia, Petzen N, Obere Krischa, 46.506N, 14.757E, 1900 m, 13.VII.2009, leg. P. Huemer, TLMF 2009-138 (TLMF); 1 female, Carinthia, Karawanken, 28.VII.1971, leg. Wettstein J. (HNHM); 1 male, Carinthia, Emberger Alm, Nassfeldriegel, 1920 m, 26.VII.2013. leg. C. Wieser (LMK); 1 male, Carinthia, Lesachtal, St. Jakob, Mussen, ca 1800 m, 46°42'42"N, 12°55'55"E, 24–25.VII.2000, leg. P. Huemer & S. Erlebach, TLMF 2000-01, slide No. OP1415m, BC TLMF Lep 04569 (barcode) (TLMF); 1 female, same data, but BC TLMF Lep 04569 (barcode) (TLMF); 1 male, Carinthia, Overvellach, 10.VII.1967, TLMF Innsbruck H. Kolbeck 2014-032 (TLMF); 1 female, Tyrol, Brennergebiet, Vennatal, 27.VII.1900, coll. Dr Galvagni, slide No. RL12124f (NHMW); 1 female, Tyrol, Venediger Mts., Dorfertal, Wiesenkreuz, 1520 m, 8.VII.1993, leg. P. Huemer, TLMF 2000-01, slide No. OP1416f, BC TLMF Lep 04568 (barcode) (TLMF).

**Slovenia**. 1 female, Nova Gorica, 20.VI.1979, leg. Reiser, TLMF 2014-032 (TLMF).

**Hungary**. 1 male, “Hungaria”, coll. E. Frivaldszky, No. 1383 (HNHM).

**Romania**. 2 males, Transylvania, Borszék [Borsec], 16.VII.1942, leg. Dr Vargha Gyula, slide Nos RL12118m, RL12120m (HNHM); 1 male, Transylvania, Borszék [Borsec], 13.VII.1942, leg. Dr Vargha Gyula, slide No. RL12119m (HNHM).

**Montenegro**. 1 male, Durmitor N, Velika Stuoc N, 1940–1950 m, 43°11'25"N, 19°03'26"E, 25.VII.2011, leg. G. Tarmann, TLMF 2012-002 (TLMF); 1 male, Durmitor N, Velika Stuoc W, 1730 m, 43°11'42"N, 19°02'38"E, 24.VII.2011, leg. G. Tarmann, TLMF 2012-002 (TLMF).

**North Macedonia**. 1 male, NP Mavrovo, Radika valley, near bridge, 10 km NNW Sveta Voda, 41°47'20"N, 20°32'48"E, 1290–1340 m, 1–3.VIII.2011, leg. P. Huemer & G. Tarmann, slide No. OP1419m, BC TLMF Lep 05418 (barcode) (TLMF); 1 male, NP Mavrovo, Korab, Korabska jezero, Koblino pole, 2080–2180 m, 41°46'42"N, 20°34'55"E, 28.VII–1.VIII.2011, leg. P. Huemer & G. Tarmann, slide No. OP1420m, BC TLMF Lep 05528 (barcode) (TLMF); 2 males, ditto, but 28.VII.2011 (TLMF);1 male, ditto, but 2115 m, 30+31.VII.2011 (TLMF).

**Bulgaria**. 1 male, Pirin Mts, 1700 m, 15–25.VII.1933, leg. J. Thurner (HNHM).

**Greece**. 1 male, Crete, Palaeochora Umg.1–13.V.1999, leg. J. Wimmer, TLMF Innsbruck Slg. J. Wimmer 2016-019 (TLMF).

#### Diagnosis.

The two sister species are often confusingly similar externally which has led to the late recognition of their distinctness. There are, however, certain differences in the forewing pattern and the coloration (see Figs 1–20) which help in the separation of the two species, although the specific identity of specimens is more safely determined by examination of the genitalia and/or consideration of the barcodes.

**Figures 1–10.**
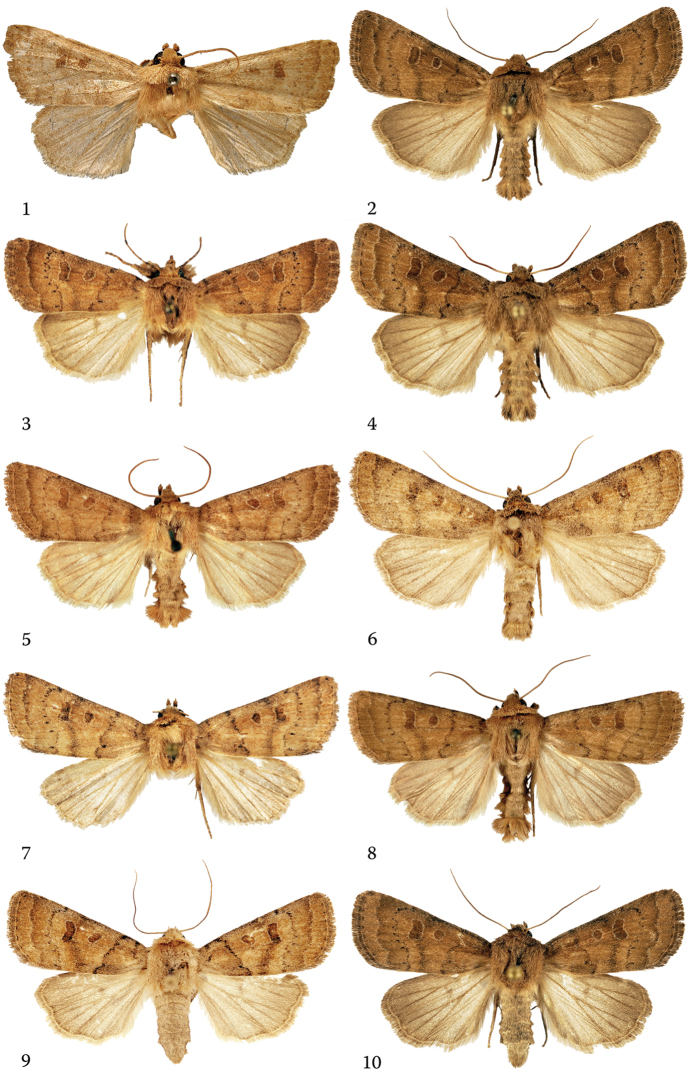
*Hoplodrina alsinides* (Costantini, 1922), adults in dorsal view. **1** Male, Lectotype of *Caradrina alsinides*, Italy, Emilia-Romagna, Sestola **2** male, Italy, South Tyrol, Sesvenna Mts. **3** male, Romania, Transylvania, slide No.: RL12119 **4** male, Italy, South Tyrol, Sesvenna Mts. **5** male, Austria **6** male, Austria, Carinthia, slide No.: OP1415, BC TLMF Lep 02471 **7** male, Italy, South Tyrol, slide No.: RL10288, BC TLMF Lep 04569 **8** male, Austria, Carinthia **9** female, BC TLMF Lep 04568 **10** emale, Italy, South Tyrol, Sesvenna Mts.

**Figure 11–20.**
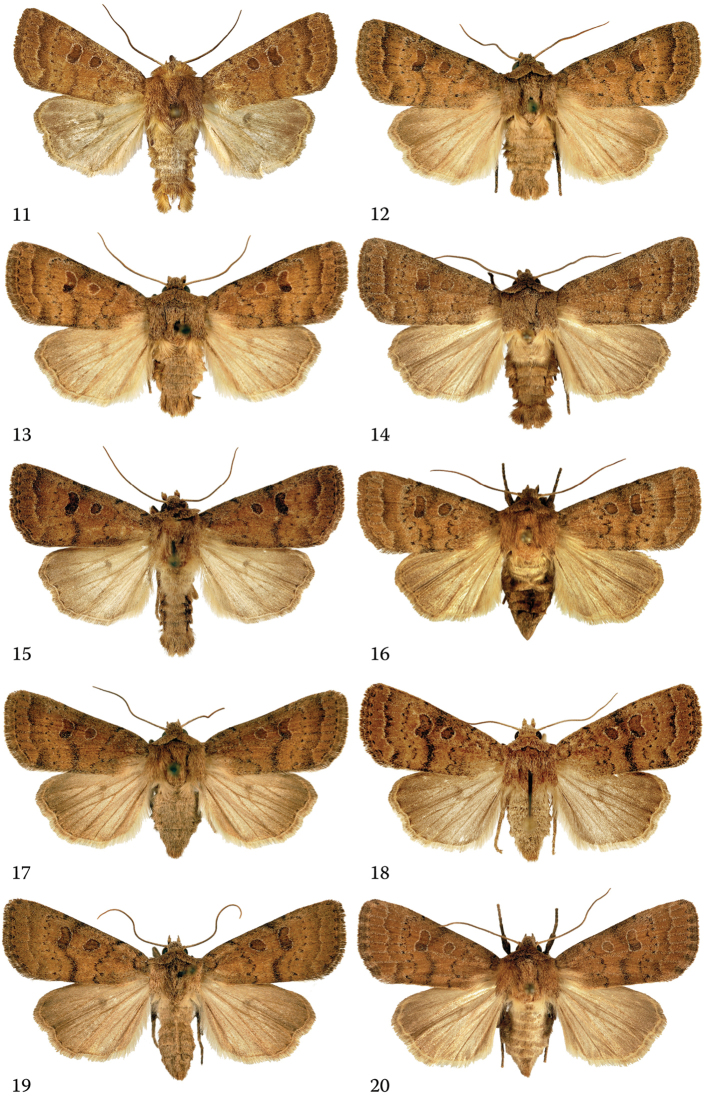
*Hoplodrina octogenaria* (Goeze, 1781), adults in dorsal view. **11** Neotype male, Germany, Bayern **12** male, Austria, Carinthia **13** male, Italy, South Tyrol, Sesvenna Mts. **14** male, Italy, South Tyrol, Sesvenna Mts. **15** male, Hungary, Vas County **16** female, Hungary, Pest County **17** female, Austria, Wien **18** female, Hungary, Pest County **19** female, Austria, Burgenland **20** female, Austria, Wien.

*Hoplodrina alsinides* can be characterised, compared with *H. octogenaria*, by its somewhat narrower and slightly more pointed forewings with smoother scaling and a finer sheen; less sinuous crenate antemedial line usually followed by a fine olive-grey or olive-brown suffusion which often extends to the entire basal area; more diffuse and weaker median fascia and less sharply marked, usually less crenate postmedial line. It is worth noting that certain *H. alsinides* specimens have darkened basal and marginal areas and a paler median field, this “trizonal” forewing coloration is typical only of the new species.

**Figures 21, 22.**
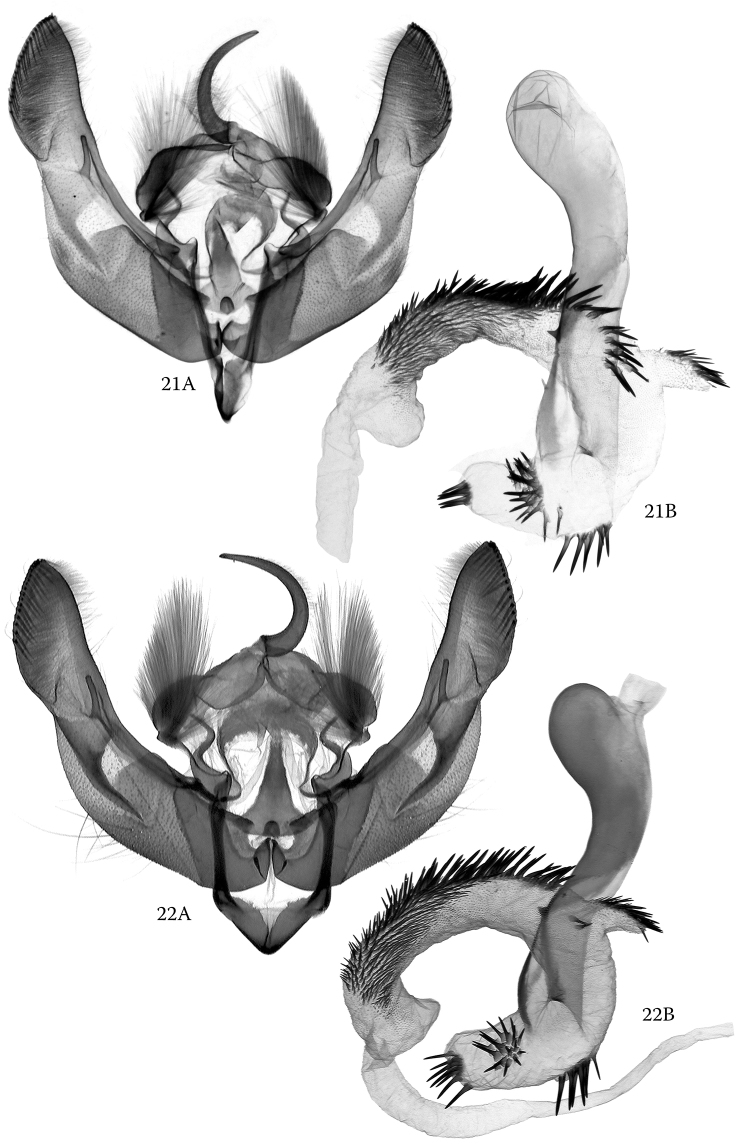
*Hoplodrina alsinides* (Costantini, 1922), male genitalia. **21A, B** Austria, Carinthia, slide No.: RL10289 **22A, B** North Macedonia, slide No.: OP1419.

**Figures 23, 24.**
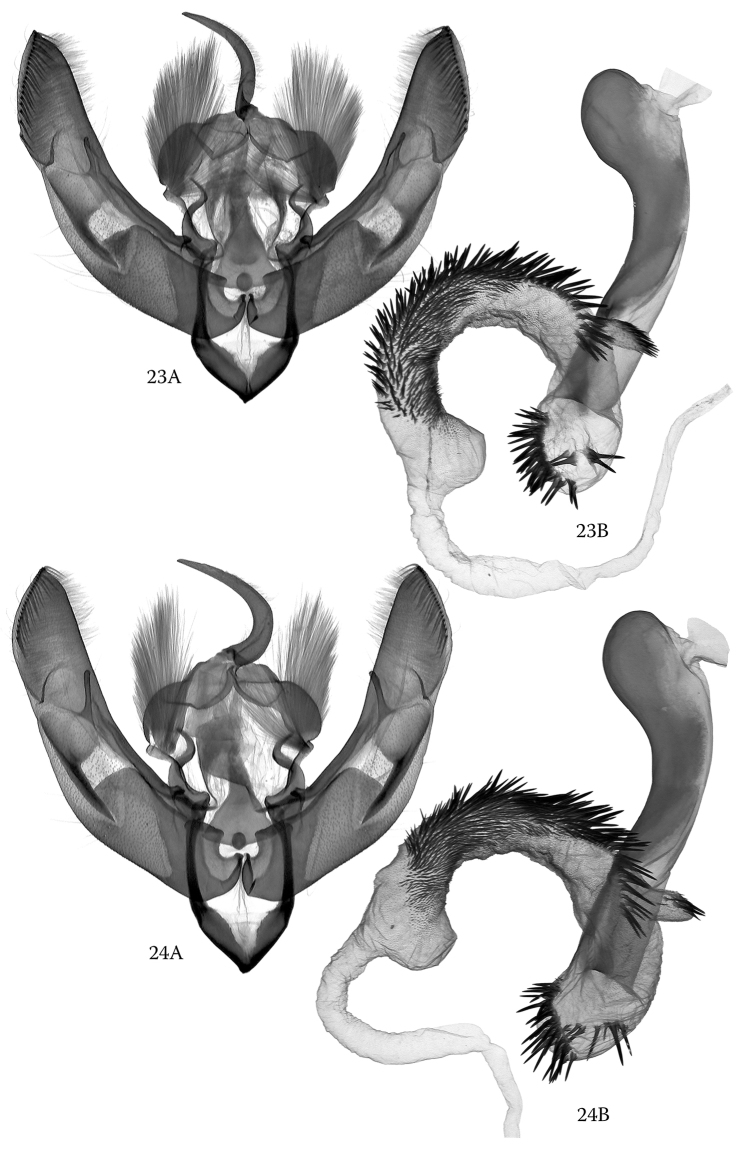
*Hoplodrina octogenaria* (Goeze, 1781), male genitalia. **23A, B** Austria, North Tyrol, slide No.: OP1407 **24A, B** Italy, South Tyrol, slide No.: OP1412.

The male genitalia of *H. alsinides* (Figs 21, 22) can be best distinguished from those of *H. octogenaria* (Figs 23, 24) by features of the vesica though the clasping apparatus also show diagnostic characteristics. The subbasal ventral diverticulum in the vesica of the new species is elongate-subconical, more elongate and narrower than in *H. octogenaria* and provided with three distinctly arranged groups of spiniform cornuti. This part of the vesica is shorter and broader and rather globular in *H. octogenaria* and provided with two longer groups of cornuti arranged in an oblique T-shaped structure. In the clasping apparatus, *H. alsinides* has, in comparison with *H. octogenaria*, proportionally shorter valvae with a stronger constriction below the cucullus, shorter and somewhat straighter (usually less arched) ampulla and a distinctly narrower subdeltoid juxta with a narrower basal section and more evenly tapering medial and distal parts.

**Figures 25–28.**
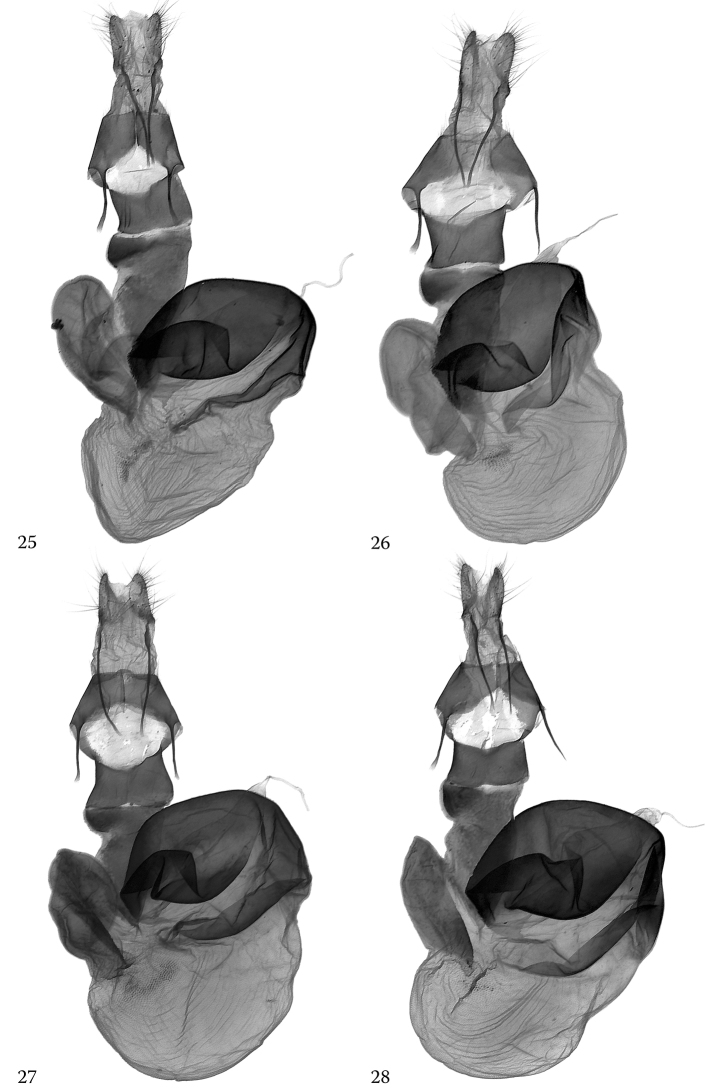
*Hoplodrina* spp., female genitalia. **25***Hoplodrina alsinides* (Costantini, 1922), Paratype, Italy, slide No.: OP1414 **26***Hoplodrina alsinides* (Costantini, 1922), Austria, East Tyrol, slide No.: OP1416 **27***Hoplodrina octogenaria* (Goeze, 1781), Austria, North Tyrol, slide No.: OP1409 **28***Hoplodrina octogenaria* (Goeze, 1781), North Macedonia, slide No.: OP1410.

In the female genitalia, the antrum of *H. alsinides* (Figs 25, 26) is more quadrangular than in *H. octogenaria* (Figs 27, 28) with rather straight lateral sides and a less dilate anterior (proximal) part, the anterior (proximal) two-thirds of the ductus bursae and the lateral appendage of the corpus bursae located opposite the appendix bursae is narrower and the corpus bursae is smaller than in its sister species. The sclerotized distal half of the last sternite is distinctly rounded triangular in the new species, being distinctly narrower, more triangular than in *H. octogenaria* which has a broader, more trapezoidal sclerotization.

#### Re-description.

Wingspan 27–34 mm. Sexes generally similar though the females are somewhat smaller and more narrow-winged than the males and there are slight differences also in the thickness of the antennae.

***Head.*** Unicolorous brown; palpi short, upturned with short third segment, sides darker brown, often greyish; frons smooth, slightly prominent, covered with long hair-scales; antennae of both sexes filiform, those of males somewhat thicker, with longer fasciculate cilia.

***Thorax.*** Usually various shades of unicolorous brown, from pale ochreous brown to deep hazel-brown mixed with whitish hair-scales; collar large, unicolorous; tegulae rather narrow; pro- and metathoracic tufts large. Legs with long brown femoral fringes.

***Forewing.*** Elongate-triangular, with finely pointed apex; ground colour matching the thorax, variable from pale ochreous brown to hazel-brown, basal and marginal areas often somewhat (or more) darker than median area; subbasal line rudimentary, dark grey; antemedial line oblique, slightly sinuous, dark grey, edged with a variably broad darker brownish or brown-grey zone on inner side; median fascia darker grey-brown, often diffuse or indistinct; postmedial line also less sharply marked, sinuous, with fine dark grey spots and streaks on veins; subterminal line pale ochreous brown, more or less straight, edged darker brown on inner side; terminal line narrow, ochreous white, marked by variably strong blackish grey dots or triangles between veins; fringes as ground colour, usually with poorly visible ochreous brown streaks at veins; orbicular stigma small, rounded or flattened, reniform stigma usually narrowly bean-shaped, both stigmata darker brown than ground colour, outlined ochreous brown; claviform stigma absent.

***Hindwing.*** Evenly rounded, apex and tornus with minute peaks only; ground colour whitish ochre, strongly suffused dark ochreous brown to grey-brown; marginal area relatively wide, darker than other parts of wing, widest at apex and tapering towards tornal angle; transverse line absent; discal spot clearly visible but diffuse, darker brown, rounded or slightly streak-like; veins and terminal line darker brown; fringes ochreous brown with darker inner line.

***Abdomen.*** Male abdomen long and slender, similar in colour to that of thorax, dorsum sometimes slightly paler, especially on segments A_1_ and A_2_; dorsal crest absent; lateral fringes and anal tuft well-developed. Female abdomen shorter and thicker, with shorter and smoother pubescence on dorsal surface; lateral fringes reduced or very short; final segment elongate, darker in colour.

***Male genitalia.*** Clasping apparatus sclerotized, relatively large. Uncus strong, curved and apically acute; tegumen broad and rather short, with well-developed, rounded and densely hairy penicular lobes; juxta narrow, subdeltoid with evenly tapering dorsal and moderately wide basal (ventral) parts; vinculum sclerotized, broadly V-shaped. Valvae symmetrical, elongate and almost evenly wide, slightly constricted below cucullus; sacculus sclerotized, long, clavus with stronger sclerotized and wrinkled setose surface; harpe (clasper) flattened, its basal part bar-like, more or less straight, apical (distal) part dilated, flattened; ampulla relatively short, thin, digitiform, straight or slightly curved; cucullus long, more or less helmet-like with acute apex, well-developed, long corona and small, triangular antero-ventral lobe. Phallus medium-long, strong, thick and arcuate, with broader, proximally evenly rounded coecum; ventral carinal plate sclerotized, long, beak-shaped, with eversible, long carinal extension. Vesica broadly tubular, everted forward, producing large, subconical subbasal diverticulum provided with three distinct groups of long spiniform cornuti; main tube of vesica bent ventrad from base of subbasal diverticulum and somewhat recurved dorsally; most of main tube densely covered with short and medium-long, strong, spiniform cornuti, its basal section with short, tubular diverticulum terminating in a bundle of fine spinules; terminal diverticulum large, subconical, membranous, without cornuti.

***Female genitalia.*** Ovipositor medium-long, conical, papillae anales elongate, apically finely rounded, sparsely setose with long sensory setae; both pairs of gonapophyses narrow, long; antrum quadrangular, flattened and sclerotized, its lateral edges more or less straight; posterior margin with shallow, arcuate cleft; ductus bursae medium-long, its posterior (distal) part somewhat broader than antrum, with rounded and sclerotized lateral lobe at right side; anterior (proximal) section of ductus bursae distinctly narrower, flattened and partly sclerotized; appendix bursae large, elliptical, partly twisted and heavily sclerotized; corpus bursae discoid-globular, membranous, wrinkled, with subconical gelatinous appendage at junction of ductus bursae and with a diffuse, irregularly rounded signum patch.

***Last abdominal segment.*** Tergite VIII very broad, quadrangular, with rounded trapezoidal, homogeneous sclerotization; sternite VIII much smaller, rounded triangular with subconical sclerotized posterior half with heavily sclerotized apical section.

#### Bionomics.

The species inhabits dry and warm, open or lightly wooded mixed forests, rocky slopes, also in open upper forest regions or even in the timberline; between altitudes of ca 1000–2200 m, according to the confirmed records. Univoltine summer species, the moths are on the wing in July and August. The early stages and the foodplant are considered to be unknown due to the uncertainty of the identification of the reared adults. As the two sister species can be found at the same site, their bionomics is presumed to be at least partly similar.

#### Distribution.

**Figure 29.**
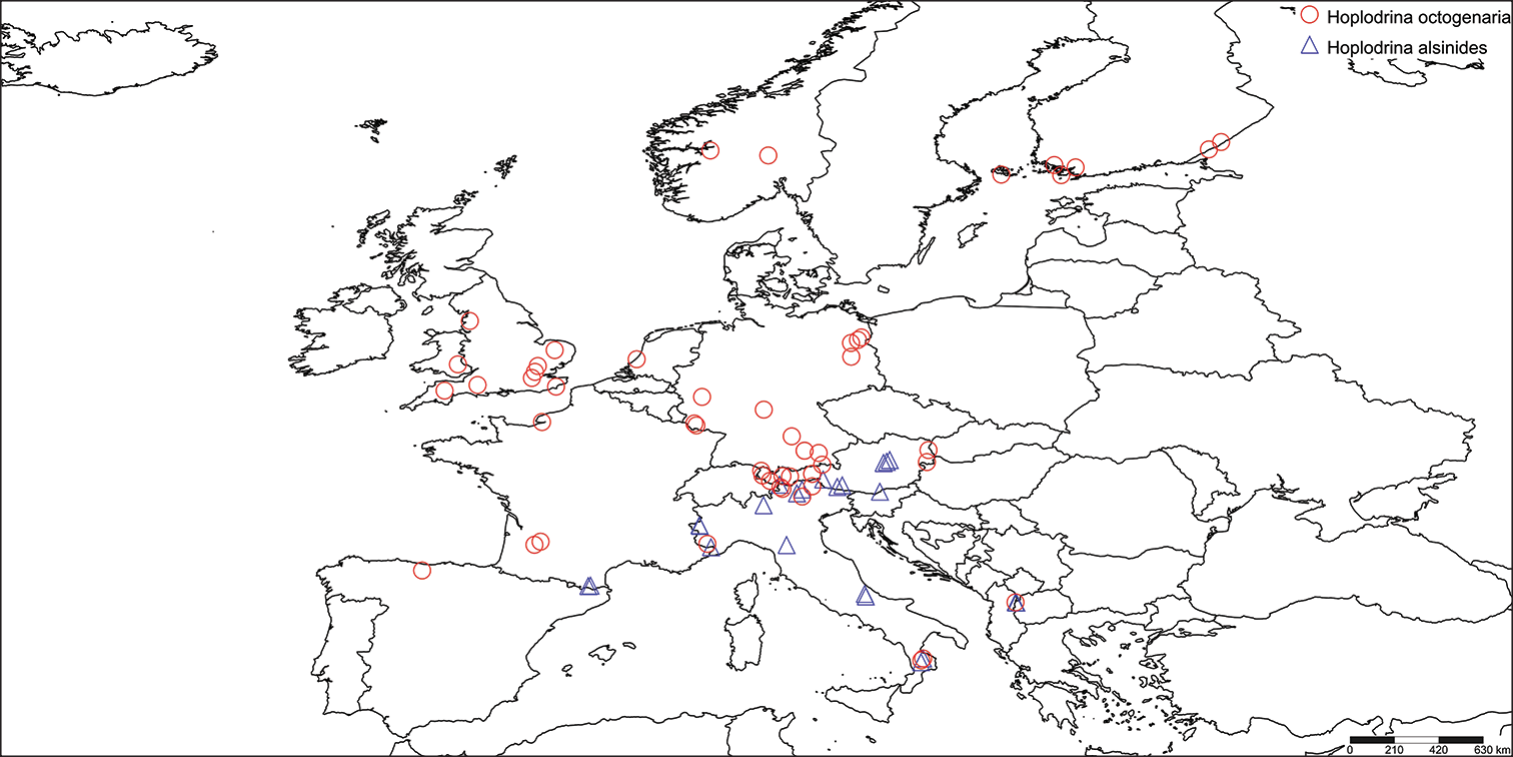
The distribution of *H. alsinides* (Cost antini, 1922) and *H. octogenaria* (Goeze, 1781) from successfully sequenced specimens. Map created with SimpleMappr (http://www.simplemappr.net).

From our proved molecular data (Fig. 29) and morphologically verified specimens, *H. alsinides* is widely distributed in southern Central Europe and the Mediterranean, ranging from southern Greece (Crete) across the Balkan Peninsula to the southern part of the Alps and the north-eastern part of the Iberian Peninsula (Spain: Aragon). It furthermore occurs on the Italian Peninsula, ranging from South Tyrol in the North to Calabria in the South and probably in most of the country. Further records are from the French Alps and Pyrenees, and from southern and eastern Austria. On some occasions the species has been found to be sympatric with *H. octogenaria*. The latter species generally has a much wider distribution with numerous confirmed records in large parts of central and northern Europe, extending to northern Spain and the British Isles in the West and Finland in the North-East, but also present in the mountain parts of the Mediterranean, e.g., southern Italy (Calabria) and Macedonia.

## Molecular results

### DNA barcode sequences

We obtained and eventually analysed 235 full length barcode sequences of 658 bp and 33 sequences ranging between 571 and 657 bp and covering all of the currently reported seven European species of *Hoplodrina*.

Nearest Neighbour distance analysis of *Hoplodrina* under the K2P model resulted in a minimum distance of 3.14% (mean 3.65%, maximum 5.25%, SE = 0.1) (Table 1) whereas the mean intraspecific distance was only 0.22%.

*Hoplodrina alsinides* clusters in a single BIN: BOLD: AAB4765 (Ratnasingham and Hebert 2013). The intraspecific distances of the barcode region are very low with 0.11% on average and a maximum of 0.35% (p-dist) (*N* = 29) whereas the minimum distance to the nearest neighbour *H. octogenaria* is 3.53%.

**Table 1.** KP2 intra- and maximum interspecific distances (in %) in European species of *Hoplodrina*.

Species	Mean Intra-Sp	Max Intra-Sp	Nearest Species	Distance to NN
*Hoplodrina ambigua*	0.1	0.66	*Hoplodrina octogenaria*	5.25
*Hoplodrina blanda*	0.38	0.96	*Hoplodrina octogenaria*	3.24
*Hoplodrina hesperica*	N/A	0	*Hoplodrina octogenaria*	3.14
*Hoplodrina octogenaria*	0.17	1.4	*Hoplodrina hesperica*	3.14
*Hoplodrina respersa*	0.24	0.62	*Hoplodrina octogenaria*	3.46
*Hoplodrina alsinides*	0.11	0.35	*Hoplodrina octogenaria*	3.82
*Hoplodrina superstes*	0.55	1.24	*Hoplodrina octogenaria*	3.46

### Data exploration, phylogenetic analyses, and SNP identification based on genome-wide SNP data

We generated a genome-wide SNP data set from 13 individuals of *Hoplodrina octogenaria* and *H. alsinides* using ddRAD sequencing, and used this data set to perform phylogenetic analyses. We obtained 2.15 million reads per individual on average, of which 1.49 million reads per individual (78.1%) were retained after quality filtering steps (Table 2). After filtering and clustering at 85% sequence similarity, we recovered 7307 putative orthologues shared across more than two samples, for a total length of 1363,146 bp. This data includes 47,676 SNPs, of which 7106 are parsimoniously informative (PIS).

**Table 2.** Summary of ddRAD data.

Species	Sample ID	Total reads (× 10^6^)	Reads passed filter (× 10^6^)	Clusters at 85%	Clusters coverage	Heterozygosity	Retained loci	Loci in assembly
*H. octogenaria*	BC ZSM Lep 82261	3.28	2.80	13514	81.4	0.001977	4711	2835
	MM07463	2.43	2.09	11050	73.3	0.001355	4499	2634
	TLMF Lep 08140	1.58	1.33	17082	33.1	0.002595	6399	3869
	TLMF Lep 10517	2.04	1.73	16433	48.9	0.002743	6347	3859
	TLMF Lep 10569	2.43	2.01	7293	57.5	0.002009	1720	918
	TLMF Lep 10642	1.54	1.18	7764	72.4	0.001191	2131	1233
	TLMF Lep 10690	5.47	0.46	12914	172.2	0.002002	4138	2459
	TLMF Lep 10804	2.30	1.90	22038	43.2	0.002218	8718	4023
*H. alsinides*	TLMF Lep 02472	2.54	2.13	20286	34.9	0.001155	6018	1525
	TLMF Lep 05418	0.76	0.64	6784	42.1	0.001085	1634	907
	TLMF Lep 05904	1.92	1.62	3381	230.8	0.000865	723	302
	TLMF Lep 05905	0.93	0.81	3636	59.8	0.000753	715	344
	TLMF Lep 13128	0.72	0.62	7438	30.8	0.000480	1018	304
	**Average**	**2.15**	**1.49**	**11509**	**75.4**	**0.001571**	**3752**	**1939**

**Figure 30.**
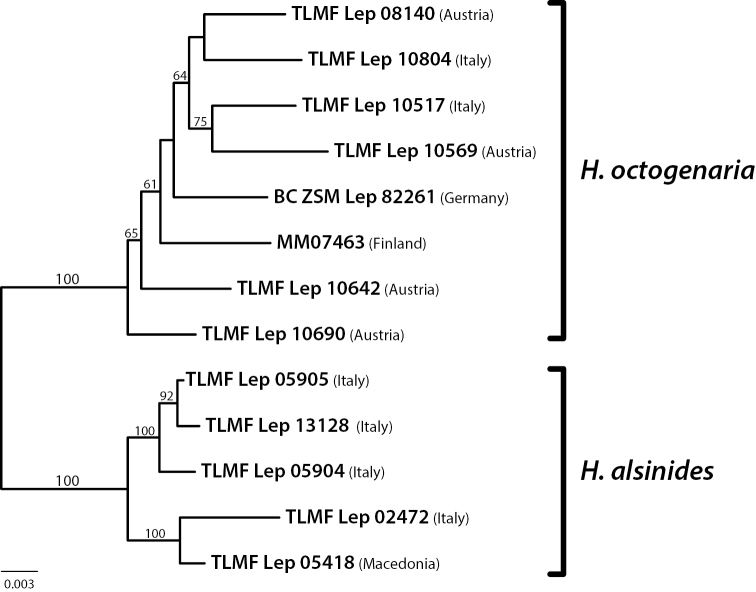
Maximum likelihood phylogeny inferred from the concatenated RAD data. The data matrix consisted of 47,676 SNPs in 1,363,146 bp. The phylogenetic tree was inferred with RAxML with 1,000 bootstrap replicates. Bootstrap support values are indicated above the branch, values of only > 50% are shown.

Phylogenetic analysis using the concatenated ddRAD dataset produced robust support for the relationship between the individuals (Fig. 30). In the ML tree, the two revealed lineages correspond to *H. octogenaria* and *H. alsinides* that were supported by 100% bootstrap support values.

**Figure 31.**
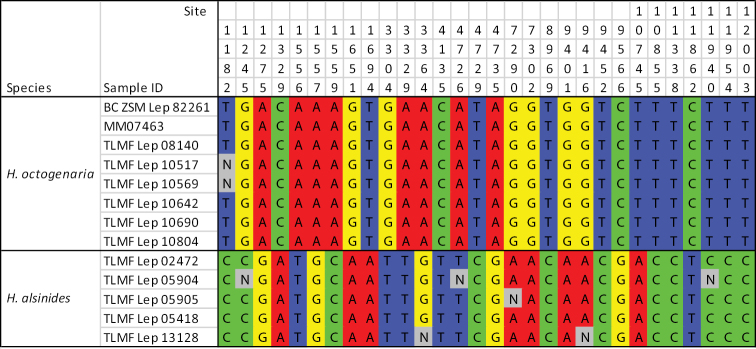
The SNPs showing a fixed difference between *H. octogenaria* and *H. alsinides*.

A total of 66 putative RAD loci had exactly one bi-allelic putative SNP and were genotyped in all 13 individuals of two species. The data includes a total of 143 SNPs, of which 61 are PIS. The SNPs occurs at 2.17 SNP/locus on average. Of these, we identified 30 fixed differences between *H. octogenaria* and *H. alsinides* sp. rev. providing candidate species-specific SNPs (Fig. 31).

Overall, the massive genomic ddRAD sequencing data provided very strong evidence that the two partially sympatric species of *Hoplodrina* are biologically distinct species.

## Supplementary Material

XML Treatment for Hoplodrina octogenaria

XML Treatment for Hoplodrina alsinides
